# Molecular Dynamics Simulation Reveals Insights into the Mechanism of Unfolding by the A130T/V Mutations within the MID1 Zinc-Binding Bbox1 Domain

**DOI:** 10.1371/journal.pone.0124377

**Published:** 2015-04-13

**Authors:** Yunjie Zhao, Chen Zeng, Michael A. Massiah

**Affiliations:** 1 Department of Physics, The George Washington University, Washington, District of Columbia, United States of America; 2 Department of Physics, Huazhong University of Science and Technology, Wuhan, Hubei, China; 3 Department of Chemistry, The George Washington University, Washington, District of Columbia, United States of America; Loyola University Chicago, Stritch School of Medicine, UNITED STATES

## Abstract

The zinc-binding Bbox1 domain in protein MID1, a member of the TRIM family of proteins, facilitates the ubiquitination of the catalytic subunit of protein phosphatase 2A and alpha4, a protein regulator of PP2A. The natural mutation of residue A130 to a valine or threonine disrupts substrate recognition and catalysis. While NMR data revealed the A130T mutant Bbox1 domain failed to coordinate both structurally essential zinc ions and resulted in an unfolded structure, the unfolding mechanism is unknown. Principle component analysis revealed that residue A130 served as a hinge point between the structured β-strand-turn-β-strand (β-turn-β) and the lasso-like loop sub-structures that constitute loop1 of the ββα-RING fold that the Bbox1 domain adopts. Backbone RMSD data indicate significant flexibility and departure from the native structure within the first 5 ns of the molecular dynamics (MD) simulation for the A130V mutant (>6 Å) and after 30 ns for A130T mutant (>6 Å). Overall RMSF values were higher for the mutant structures and showed increased flexibility around residues 125 and 155, regions with zinc-coordinating residues. Simulated pKa values of the sulfhydryl group of C142 located near A130 suggested an increased in value to ~9.0, paralleling the increase in the apparent dielectric constants for the small cavity near residue A130. Protonation of the sulfhydryl group would disrupt zinc-coordination, directly contributing to unfolding of the Bbox1. Together, the increased motion of residues of loop 1, which contains four of the six zinc-binding cysteine residues, and the increased pKa of C142 could destabilize the structure of the zinc-coordinating residues and contribute to the unfolding.

## Introduction

MID1 is a tripartite motif (TRIM) protein consisting of N-terminal RING, Bbox1 and Bbox2 domains [1]. MID1 catalyzes the polyubiquitination of the catalytic subunit of protein phosphatase 2A (PP2Ac) and alpha4 [2,3]. PP2A is a heterotrimeric Ser/Thr phosphatase complex that regulates essential cellular pathways affecting metabolism, cell-cycle progression, and apoptosis [4–15]. Alpha4 is a protein regulator of PP2A in numerous pathways including the target of rapamycin (TOR) [16–18]. Mutations of MID1 are associated with X-linked Opitz G Syndrome (XLOS), which is characterized with midline anomalies that include cleft lip/palate, hypertelorism, and hyperspadias [15,19–22].

The MID1 Bbox1 domain is required for the ubiquitination of PP2Ac and alpha4[2]. In some patients with XLOS, residue A130 is mutated to a threonine or valine [3]. Sequence alignment of Bbox1 domains, specifically within the C1 class of the TRIM protein family reveals that alanine at position 130 is highly conserved [23].

**Fig 1:**
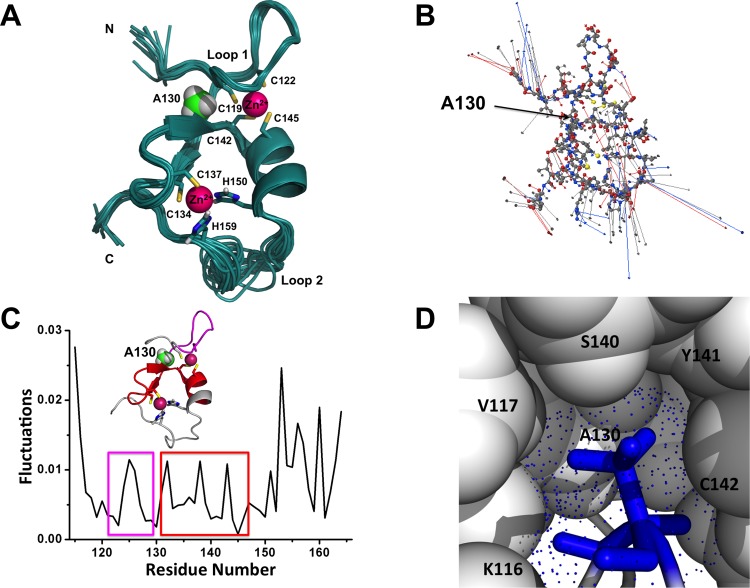
Structural properties of the MID1 Bbox1 domain. (A) The ensemble of 13 structures generated from NMR-derived restraints (PDB code: 2FFW). (B) Magnitude of the fluctuation represented as eigenvectors of the Bbox1 ensemble of structures. The conformational fluctuations indicate both magnitude and directions (arrows) and are derived from PCA analysis. (C) The fluctuations of the average NMR structure are shown as a function of residue number. (D) Zoomed-in snapshot of the small cavity near residue A130. The blue dots represent the solvent accessible surface.

The Bbox1 domain is a small zinc-finger domain, of approximately 60 amino acids, that coordinates two zinc ions in a cross-brace fashion and adopts a tertiary structure similar to the ββα-RING folds of ubiquitin E3 ligases (Fig 1A). The structure contains two loops, one of which is well defined (residues 118–129) and contains the lasso-like loop (residue 118–141) and a β-strand–type II turn–β-strand (β-turn-β) substructures. The structure reveals that residue A130 is located on loop 1 at a point that separates the lasso-like loop and β-turn-β sub-structures (Fig 1A) [1]. Even though the methyl group of A130 does not make extensive hydrophobic contacts with other residues, its relative position in the ensemble of NMR structures is rather fixed (Fig 1A). The shorter loop 2 consists of residues 150–159 which includes H157 that coordinates the second zinc ion. The structure of residues 151 to 156 is less defined due to their mobility.

Two-dimensional ^1^H-^15^N HSQC NMR studies of the MID1 Bbox1 domain with the A130T mutation showed a spectrum with significant collapse in the dispersion of its cross peaks compared to the spectrum of the native protein. The similarity of the poorly dispersed spectra of the A130T mutant Bbox1 protein and the native Bbox1 protein in the presence of 10x molar excess of ethylenediaminetetraacetic acid, a strong metal chelator, indicated that the coordination of both zinc ions was lost with the mutant [24]. While the NMR studies can reveal the effect of the A130T mutation on the structure, it cannot provide insights into why or how the mutation would cause the structure of the Bbox1 domain to unfold.

MD simulations can be effective in understanding how the two mutations at position 130 perturb the protein structure. The accuracy in computational modeling and MD simulation has made it possible to study processes that are too fast to be observed experimentally [25–27]. These approaches have been successfully employed to address questions ranging from protein folding to drug design and screening [25,28–30].

In this article, MD simulation analyses of the two A130T/V mutations revealed that residue A130 served as a hinge point and its mutation caused steric clashes that in turn increased the dielectric constant in one of the zinc-binding regions. The increased motion of residues on loop 1 disrupts the coordination of both zinc ions that are 13 Å apart.

## Methods and Materials

### Principal component analysis

Structures used in the principal component analysis (PCA) were experimentally determined by NMR spectroscopy [1]. The direction and magnitude of the relative motion of each residue were calculated by the PCA_NEST program [31]. PCA_NEST is designed to extract the principal modes of conformational changes from an ensemble of structures. The structures were first optimally superimposed by iterative best-fitting approaches using the Kabsch algorithm [32], and then followed by PCA. Conformational changes and structural motions were visualized and analyzed by the Jmol visualization package (http://www.jmol.org/).

### Molecular dynamics simulations

All simulations on the native MID1 Bbox1 domain and specific mutations were performed using the GROMACS software package [33]. The calculations of the native Bbox1 domain used model 1 of the NMR ensemble of structures (PDB code 2FFW) [1]. The mutant structures were built via homology modeling by changing residue A130 to threonine or valine using I-TASSER [34]. For the simulation, the Amber03 force field [35] and explicit solvent based on the TIP3P model were employed [36]. The structures were solvated with explicit water in the periodic rectangular boxes under normal (150 mM) saline condition. The LINCS algorithm was used to constrain all bond lengths [37]. Long-range electrostatic interactions were treated with the particle-mesh Ewald method [38]. For the MD calculations, the non-bonded (electrostatic and VDW) cutoff range was 8 Å and the time step was 2 fs.

Before each MD simulation, the entire system was first minimized by a 1000-step steepest descent calculation followed by a 3000-step of conjugate gradient optimization. We performed three 100 ns-trajectories at 300K for each of the native and two mutant structures, for a total 900ns computer simulation time. The standard deviation (SD) of backbone RMSD among the three repeated MD trajectories for the native, and the A130T/V mutants are 0.2Å, 0.7Å and 0.8Å, respectively. The small SD values (<1Å) indicate that the simulated trajectories are consistent. The pattern of the secondary structure elements were calculated using standard DSSP program [39]. The structures were visualized and analyzed by VMD and PyMOL [40]. The pKa values for residue C142 were calculated by PROPKA [41,42]. The root mean square deviation (RMSD), root mean square fluctuation (RMSF), and angles were carried out by using the gromacs inbuilt tools g_rms, g_rmsf and g_sgangle, respectively.

## Results

MID1 Bbox1 domain coordinates two zinc ions, one with four cysteine residues (C119, C122, C142, C145) and the other with two cysteine residues (C134, C137) and two histidine residues (H150, H157) (Fig 1A) [1]. We recently showed that mutations of residues C142 and C145 resulted in loss of coordination of both zinc ions and unfolding of the Bbox1 domain (see Figs 1–4 in Wright et al. [24]). The NMR spectrum for the native Bbox1 domain showed well-dispersed signals in the two-dimensional ^1^H-^15^N HSQC spectrum. Even though residue A130 is not involved in zinc coordination, its mutation disrupts the coordination of both zinc ions (see Fig 4 of ref [24]). The ^1^H-^15^N HSQC spectrum of the A130T mutant Bbox1 domain showed ^15^N–H cross peaks with very little chemical shift dispersion in both the NH and ^15^N dimensions. The spectrum of the A130T mutant Bbox1 domain is similar to that of the C142S mutant Bbox1 domain and native Bbox1 in the presence of EDTA, a potent divalent metal chelator that would remove the coordinated zinc ions [24]. While the NMR spectrum of the A130T mutant Bbox1 protein indicated that the resultant protein structure is unfolded, it is unclear how the mutation to valine or threonine would induce or influence unfolding of the structure. To gain insights into the mechanism of how the A130T/V mutant could affect the structure, we evaluated the properties of the NMR ensemble of structures and performed MD simulations.

### Structural features of the Bbox1 protein

Before subjecting the Bbox1 domain to MD simulation studies, we evaluated its structural features by extracting the pattern of motions for each amino acid from the ensemble of NMR structures using the PCA method. Superposition of backbone atoms of 13 NMR structures (PDB accession code 2FFW) showed good precision for most residues except for residues of loop 2 and the N-terminal region that encompasses the lasso-like loop (Fig 1A). The PCA-defined conformational fluctuations favored by model 1 within the ensemble of the structures correlated well with the level of precision with the ensemble. The direction of motion and the magnitude of the fluctuation are represented by eigenvectors depicted in Fig 1B as blue and red lines for backbone N and O atoms, respectively. Since the length of an eigenvector is proportional to the magnitude of the fluctuations, Fig 1B reveal that the residues on outer regions of the structure are much more flexible than the ones near the core. Next, we measured the differences of eigenvalues obtained from singular value decomposition of all-atom-based covariance within the NMR ensemble of structures (Fig 1C) [31]. Higher values indicate greater differences between any two structures, whereas values of approximately zero indicate structural similarity. The analysis showed large variations of eigenvalues at the termini, with values greater than 0.02. Residues within the structured portion of the Bbox1 domain show smaller variations of eigenvalues that are less than 0.005 indicating greater stability. Small eigenvalues for specific residue or region might suggest that these residues have a greater contribution to structural stability or have a functional role. Consistent with this observation, the eigenvalue of A130 was 0.002 while adjacent residues have larger eigenvalues (Fig 1C). These results indicate that residue A130 may be located at a point on loop 1 that separates two adjacent sub-structures consisting of the β strand–turn–β strand and lasso-like loop.

**Fig 2:**
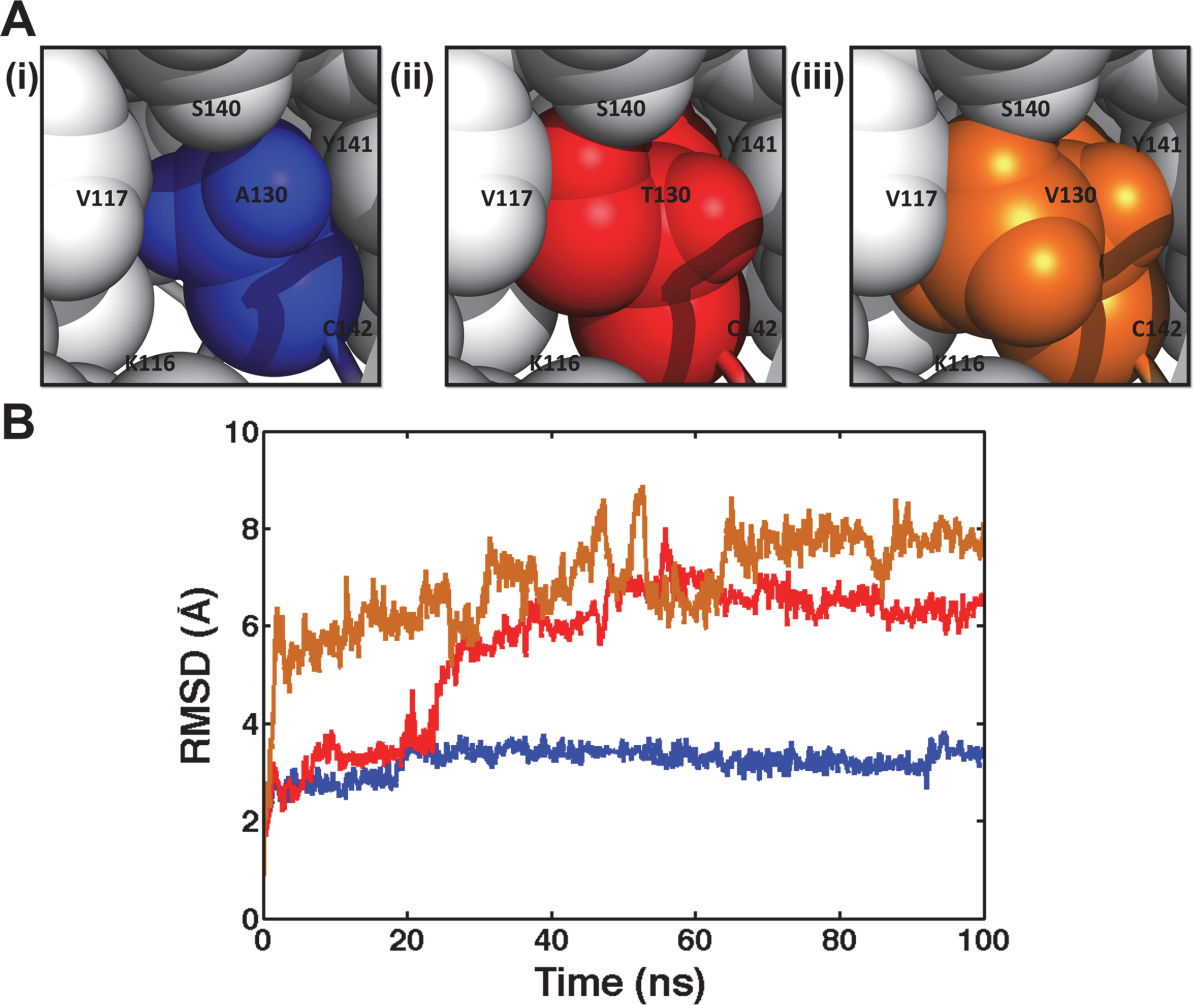
Effects of mutating of residue A130. (A) Homology modeling of Bbox1 structures highlighting the position and packing of side chain of A130 (i) compared to those of the T130 (ii) and V130 (iii). (B) Backbone RMSDs for WT (blue), A130T (red), and A130V (orange) during the MD simulations.

The alanine at position 130 is highly conserved among Bbox1 domains based on sequence alignment [3]. To evaluate the solvent accessible surface around the region of A130, we used a water sphere, with a radius of 1.4 Å, to search for accessible space in which water (blue dots) could freely occupy. A snapshot of this search revealed that the side chain of A130 points towards the inside of the protein and packs against the side chains of residues V117, C119, T133, S140 and C142 (Figs 1D and 2A). The analyses indicate that the limited accessible space that the methyl group of A130 occupies is not sufficient to easily accommodate a larger side chain. Together, these results suggest that A130 may be located at a hinge site within the Bbox1 domain, with a suitable size and chemical property to allow only a limited range in the degree of rotation between adjacent residues.

### MD simulations revealed changes in structural topology

In light of these results, we next performed large-scale MD simulations to investigate the structural consequences of mutating residue A130 to threonine or valine, which are two naturally occurring mutations observed in patients with XLOS [3]. Fig 2A shows the structures in which the alanine at position 130 is mutated to a threonine and valine based on homology modeling.

To evaluate variations in the residue positions of the Bbox1 domain, RMSD values of the position differences of backbone atoms between the mutant and native structures were calculated throughout the MD simulations (Fig 2B). The native and A130T mutant Bbox1 domains show similar deviations from their starting structures during the first 20 ns of the trajectory. After 30 ns, the structure of the A130T mutant shows a significant increase in deviation to ~6 Å, which at this point represented a non-native structure. For the structure of the A130V mutant the trajectory shows a rapid increase to 6 Å after 2 ns of the simulation run, indicating an immediate departure from its starting structure. The larger RMSD values for both mutant structures indicate that the overall topologies of the structures have changed. In contrast, the native Bbox1 protein shows a smaller RMSD or a greater stability during the entire MD simulation with the overall topology similar to the native fold. The observed RMSD of 3 Å is primarily attributed to large variations of residues at the termini and loop 2. Even though the simulation was for 100 ns, the divergence from the starting structure (or the putative native structure) for the mutant structures within the first 30 ns was sufficient to indicate that the mutant structures were substantially denatured.

**Fig 3:**
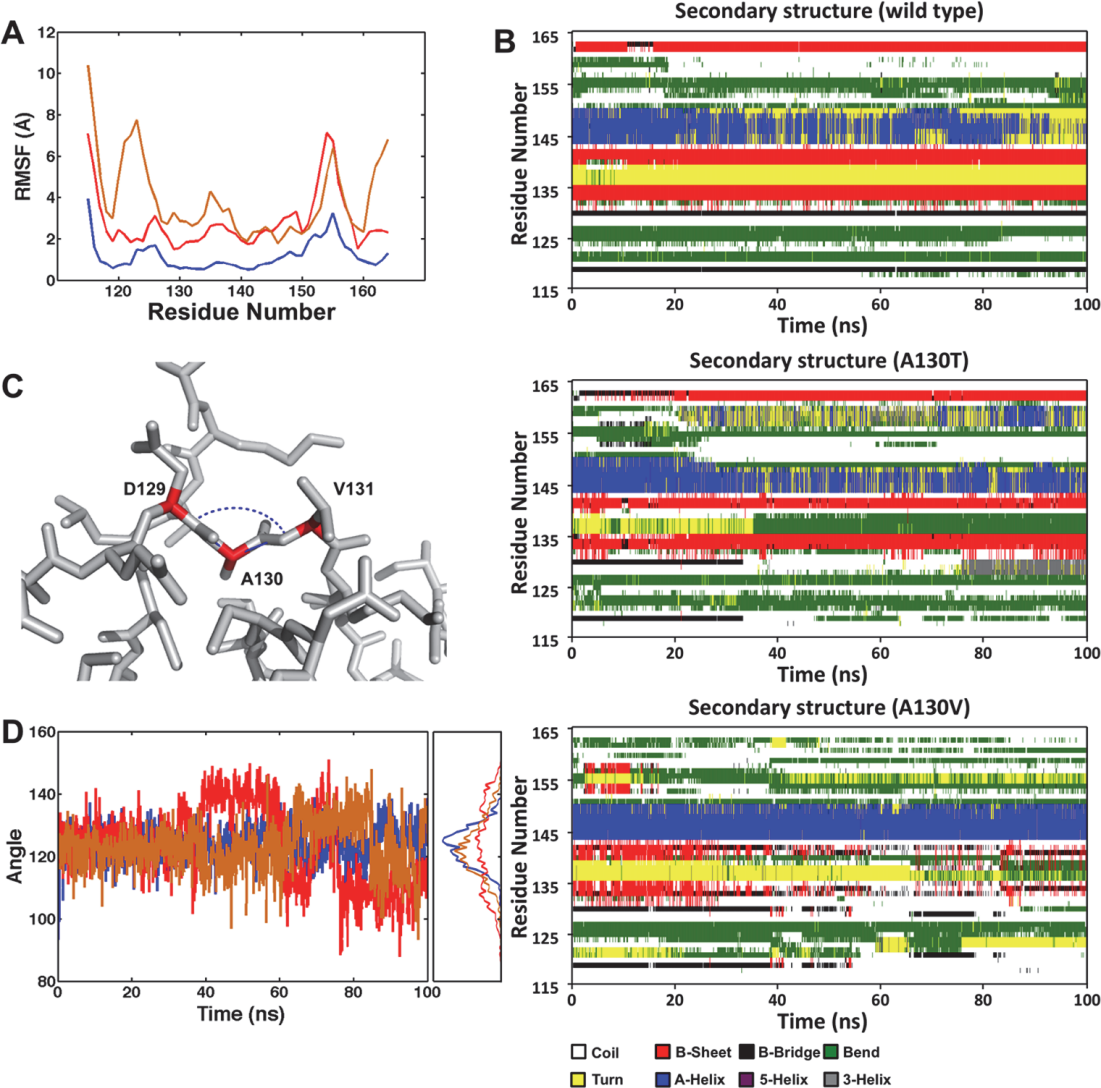
Structural analysis of the MD simulation results. (A) Backbone RMSFs for WT (blue), A130T (red), and A130V (orange) during the MD simulations. (B) Time evolution of the secondary structural elements, based on DSSP classification, of the wild type and mutant proteins. (C) The angle formed by the Cα atoms of D129, A130, and V131. (D) The angle fluctuations for WT (blue), A130T (red), and A130V (orange) during the MD simulations with the corresponding histogram to the side.

To probe how the mutations affect the dynamics of the backbone atoms, the root mean square fluctuation (RMSF) values were calculated for backbone atoms at each time point of the trajectories of the native and mutant structures (Fig 3A). Higher RMSF values indicate greater flexibility during the MD simulation. As shown in Fig 3A, the RMSF values of the A130V and A130T mutant structures are higher than those of the native structure. Similar to the RMSD results, the RMSF values for the A130V mutant show considerably higher values. In the native structure, some flexibility is observed for adjacent residues near residue D125 within loop 1. However, for A130V mutant, considerably greater flexibility for residues 120–123 including the zinc-binding C122 is observed (Fig 3A), whereas the level of flexibility for the corresponding residues within the A130T structure lies in between but closer to the native. These results suggest that the much larger side-chain of valine extends its steric hindrance to other zinc-binding residues. Supporting the increased flexibility, we also measured the RMSF values for second zinc-binding residues (C134, C137, H150, H159). Compared with the native structure, the RMSF values for regions 134–137 and 150–159 increase considerably for the A130T and A130V mutant structures, respectively, as compared with a value of 0.04 for the native structure. The larger RMSF values indicate increased random motions of these residues, particularly for the valine substitution and this could account for the loss of coordination of the second zinc ion.

Using DSSP classification of secondary structure elements, we calculated the DSSP classification for each amino acid and showed its time course in Fig 3B. The segment of residues 132–141, which serves as a bridge between two zinc-binding domains, remains as beta-turn-beta (red-yellow-red) conformation throughout the simulation, whereas it becomes beta-bend-coil-beta in A130T and coil-turn (bend)-coil in A130V. The beta sheets in this segment disappear almost completely and thus potentially destroy zinc-binding coordination. Additional conformation changes are also noted for the region of residues 150–159. Overall, we observed that about 42% of the native structure was part of secondary structures (α-helix, β-strands, and β-turn), and that percentage dropped to approximately the same 33% for both the A130T and A130V mutants (albeit with different composition in secondary structural elements). Such a reduction as well as specific conformation changes could induce the loss of the coordination of the zinc ions.

Lastly, we compared the fluctuation in the backbone position of residue 130 by calculating the angle formed between the Cα atoms of D129, V131 and residue 130 in which the alanine was mutated to valine or threonine (Fig 3C). The differences in angles associated with the A130T and A130V mutations are observed to be slightly smaller (123^°^ ± 13 and 123° ± 8, respectively), than that of A130 (124° ± 6) in >80% of the frames during the MD simulations (Fig 3D). The smaller angles and larger fluctuations indicate that the backbone atoms of residue D129 and V131 had to rotate in a way to accommodate the larger side chains of valine and threonine.

### Increased solvation contributes to higher pKa of C142

Replacing alanine with either valine or threonine positioned the methyl groups of valine and threonine by one C–C bond length closer to the side chain methylene group of C142. Residue C142 is involved in the coordination of one of the two zinc ions (Fig 1A and 1D). As a result of the amino acid change, the MD simulation shows increased backbone dynamics for adjacent and distant residues that resulted in the formation of a large opening near the side chain location of residue 130. Subsequently, the accessible surface for water molecules increases, resulting in an increase of the pKa value of the residue C142. We measured the apparent pKa values of the sulfhydryl groups of C142 for the native and two mutant structures using the empirical software PROPKA (Table 1). In order to better interpret the pKa data using the empirical method of PROPKA, we calculate the average pKa values over the multiple simulation trajectories. Whereas the average pKa value of C142 was 6.9 ± 0.6 throughout the NMR native structures, the average pKa values of C142 in the A130T and A130V mutant structures are 9.0 ± 0.5 and 9.2 ± 0.7, respectively. These increases in pKa values suggest that the thiol group of C142 would most likely be protonated, as the cavity that residue A130 occupies opens up to increase solvation, and this would prevent the sulfur from coordinating the zinc ion.

**Table 1:** Apparent average pKa values of residue C142 during the multiple MD simulations for the native protein and two mutants (A130T and A130V).

Time (ns)	C142*		
	Mutations		
	Native	A130T	A130V
5	7.6	9.4	10.7
10	7.9	10.0	9.1
15	7.9	9.5	8.6
20	6.2	9.8	9.4
25	6.2	9.7	8.7
30	6.9	8.9	10.8
35	7.0	8.9	9.9
40	6.0	8.6	8.7
45	6.9	9.4	10.0
50	6.8	8.0	8.9
55	7.4	8.6	9.5
60	7.4	8.5	8.6
65	6.7	9.2	8.9
70	6.3	8.8	8.9
75	6.2	9.0	9.5
80	7.5	8.6	8.9
85	6.7	8.3	8.3
90	7.3	9.0	8.6
95	6.3	8.4	8.8
100	7.3	8.6	8.6
Average	6.9 (±0.6)	9.0 (±0.5)	9.2 (±0.7)

* Calculations were performed in triplicates and values averaged.

## Discussion

The ββα-RING-like fold of the Bbox1 domain is essential for its function in catalyzing ubiquitin chain elongation of PP2Ac and alpha4 [3]. Natural mutations associated with the Bbox1 domain disrupt its ability to catalyze the polyubiquitination of alpha4. The mutation of residue A130 to threonine or valine causes the Bbox1 domain to unfold [24]. Unlike the mutations of the zinc-coordinating C142 and C145 that results in loss of coordination of both zinc ions and unfolding [24], it is unclear how the A130T/V mutations cause the Bbox1 domain to unfold.

With the use of MD simulation, a possible mechanism emerges showing that the slightly bulkier side chain groups of threonine and valine cause steric clashes with residue C142. The simulation revealed that while both residues affect the folding property and stability of the Bbox1 domain, the valine side chain has a more immediate and significant effect in disrupting the structure. While the RMSF and RMSD data support the notion that both substitutions destabilize the structure by increasing the flexibility of key regions of the protein, the RMSD results for the A130V substitution show a rapid increase within the first 5 ns of the simulation, indicating that the larger valine side chain has a greater structural impact than the threonine side chain. The 100 ns MD simulation was sufficiently long to show the rapid divergence from the putative native structure for the mutant structures, which occurred within the first 30 ns.

The rapid increase in flexibility is postulated to open the compactness of the structure, allowing for increased solvation within the cavity formed by residues K116, V117, A130, V131, K132, T133 and S140 that encompass residues C142 (Fig 1D). Indeed, the apparent pKa values of thiol groups of residues C142 increase to 10.5–11.5 for the two mutants within the first 30ns. The inability to coordinate one of the zinc ions destabilizes the structures of residues of loop 1 (Figs 1 and 2B), which include residues C134 and C137 that coordinate the other zinc ion. The RMSD analysis shows that the motion of the residues that coordinate the second zinc ion increases and that could account for the inability to coordinate the other zinc-ion. Thus, based on the MD simulation, we postulate that the mutation of residue A130 disrupts the coordination of the first zinc ion, coordinated by residues C119, C122, C142 and C145, followed by increased motion of residues of β strand-turn-β strand and loop 2 regions that disrupted the coordination of the second zinc ion.
